# High-Dose Testosterone Treatment Increases Serotonin Transporter Binding in Transgender People

**DOI:** 10.1016/j.biopsych.2014.09.010

**Published:** 2015-10-15

**Authors:** Georg S. Kranz, Wolfgang Wadsak, Ulrike Kaufmann, Markus Savli, Pia Baldinger, Gregor Gryglewski, Daniela Haeusler, Marie Spies, Markus Mitterhauser, Siegfried Kasper, Rupert Lanzenberger

**Affiliations:** a Department of Psychiatry and Psychotherapy; b Department of Biomedical Imaging and Image-guided Therapy, Division of Nuclear Medicine; c Department of Obstetrics and Gynecology, Medical University of Vienna, Vienna, Austria

**Keywords:** Estradiol, Hormone treatment, Positron emission tomography, Serotonin transporter, Testosterone, Transsexual

## Abstract

**Background:**

Women are two times more likely to be diagnosed with depression than men. Sex hormones modulating serotonergic transmission are proposed to partly underlie these epidemiologic findings. Here, we used the cross-sex steroid hormone treatment of transsexuals seeking sex reassignment as a model to investigate acute and chronic effects of testosterone and estradiol on serotonin reuptake transporter (SERT) binding in female-to-male and male-to-female transsexuals.

**Methods:**

Thirty-three transsexuals underwent [^11^C]DASB positron emission tomography before start of treatment, a subset of which underwent a second scan 4 weeks and a third scan 4 months after treatment start. SERT nondisplaceable binding potential was quantified in 12 regions of interest. Treatment effects were analyzed using linear mixed models. Changes of hormone plasma levels were correlated with changes in regional SERT nondisplaceable binding potential.

**Results:**

One and 4 months of androgen treatment in female-to-male transsexuals increased SERT binding in amygdala, caudate, putamen, and median raphe nucleus. SERT binding increases correlated with treatment-induced increases in testosterone levels, suggesting that testosterone increases SERT expression on the cell surface. Conversely, 4 months of antiandrogen and estrogen treatment in male-to-female transsexuals led to decreases in SERT binding in insula, anterior, and mid-cingulate cortex. Increases in estradiol levels correlated negatively with decreases in regional SERT binding_,_ indicating a protective effect of estradiol against SERT loss.

**Conclusions:**

Given the central role of the SERT in the treatment of depression and anxiety disorders, these findings may lead to new treatment modalities and expand our understanding of the mechanism of action of antidepressant treatment properties.

It is commonly suggested that the uneven sex distribution in the prevalence and clinical presentation of mood and anxiety disorders is universal. Women are approximately twice as likely to be diagnosed with depression and anxiety disorders as men (1, 2). In addition, women exhibit earlier age at onset of depression, a greater number of depressive symptoms, and a greater number of episodes (3). Several biological and psychological mechanisms potentially underlying these epidemiologic findings have been discussed. Although self-report bias, gender-role stereotypes, and sociocultural norms may confound effects of these mechanisms (1, 2), a strong case has been made for the effect of sex differences in coping styles and response to stress (4, 5), as well as in genetic and endocrine influences. Indeed, many women experience depressive symptoms in periods of hormonal fluctuations such as with menses, during and after pregnancy, or during perimenopausal transition (6, 7, 8).

Within the last 15 years, an increasing amount of molecular imaging studies using positron emission tomography (PET) have examined serotonergic key proteins such as the serotonin reuptake transporter (SERT) and serotonergic receptors in depressed versus healthy subjects (9, 10, 11). In a recent meta-analysis, we reported reduced SERT expression of about 10% in major depression and that severity of depression was associated with SERT reduction in the amygdala (12). In line with the above-mentioned epidemiologic findings, several studies also suggested sex-specific abnormalities in the expression of the SERT (13, 14, 15, 16) or the serotonin 1A receptor (17, 18). Moreover, several PET studies also indicated gender differences in regional protein binding in the healthy human brain (19, 20, 21, 22), while others found no such difference (23, 24, 25). However, whether sex hormones directly or indirectly play a role in the underlying cause of these abnormalities remains unknown.

Still, animal and human studies provided evidence for an important interplay between gonadal hormones and the serotonergic system. While human data have often applied merely correlational approaches (26, 27, 28, 29), hormone treatment of ovariectomized animals has provided convincing evidence of a more causal relationship between sex hormones and serotonergic neurotransmission (30, 31, 32, 33). Early data indicated that testosterone or estradiol treatment is associated with increased density of serotonin reuptake sites [reviewed by Bethea *et al.* (31)], a finding that has been replicated more recently and shown to be mediated by nuclear estrogen receptors (34). This is in agreement with reduced SERT density in ovariectomized mice, in which estradiol levels are low (32). In contrast, low estradiol levels in the same animals were associated with increased regional SERT activity, which, in turn, concurs with rapid reductions of 5-HT uptake in neuronal cell lines after estradiol treatment (35).

Taken together, animal studies point toward a strong influence of gonadal hormones on SERT expression and function, whereas human data are almost absent and only correlational in nature. Direct experimental approaches, in which independent variables are effectively manipulated and their impact on dependent variables is observed, have yet to be performed. Investigating the effects of chronic steroid hormone treatment in subjects with gender identity disorder provides such an experimental approach. These subjects are seeking life-long treatment with high dosages of cross-sex steroid hormones to adjust their physical appearance to their gender identity. Thus, testosterone is used when seeking masculinization (progestins are sometimes added to stop menstrual bleeding), while estrogens are administered to achieve feminization together with antiandrogens (e.g., cyproterone acetate), which block testosterone binding to androgen receptors but also show strong antigonadotrophic properties. We aimed to investigate SERT binding using PET and the radioligand [^11^C]DASB in transsexual subjects before and during cross-sex steroid hormone treatment. Based on the animal data presented above, we hypothesized that testosterone, as well as estradiol treatment, will increase SERT binding and that treatment-induced increases in hormone plasma levels will correlate with increases in SERT binding. For comparison, a group of healthy female and male control subjects receiving no treatment was further included.

## Methods And Materials

### Subjects

The study sample consisted of 33 transsexual subjects and 35 control subjects: 14 female-to-male (FtM) and 19 male-to-female (MtF) transsexuals, 11 female control subjects (FC), and 24 male control subjects (MC). Transsexuals were recruited from the Transgender outpatient unit of the Department of Obstetrics and Gynecology, Medical University of Vienna. They were naive to steroid hormone treatment and were seeking sex reassignment. Transsexuals reported gender dysphoria since a relatively early age (before or at puberty). Mean age was numerically lower in FtM (28.21 ± 6.81, mean ± SD) compared with MtF (31.79 ± 9.21), FC (30.43 ± 10.07) and MC (34.14 ± 11.00), although this difference was not significant (*p* = .38, analysis of variance). To rule out internal medicine and neurological disorders, subjects underwent a standard medical examination including electrocardiogram, routine laboratory tests, and the Structural Clinical Interview for DSM-IV Disorders. Further exclusion criteria included intake of psychotropic medication within 6 months before inclusion, past or current substance abuse, pregnancy, and hormonal treatment before study participation. Diagnostic assessment of transsexualism followed DSM-IV-TR and ICD-10 and was performed after several semi-structured, sociodemographic, clinical, and psychiatric interviews based on legal requirements for cross-sex hormonal treatment in Austria. Psychotherapy before and during hormone treatment was also recommended. After at least 1 year of hormone therapy, subjects could decide to undergo sex reassignment surgery (36). The recommendations in Austria are largely based on the Standards of Care for the Health of Transsexual, Transgender, and Gender Nonconforming People, The World Professional Association for Transgender Health, 7th Version, Atlanta, Georgia, 2011. Therefore, none of our study participants had sex reassignment surgery before or during study participation. However, two FtM and one MtF already had their official names changed at screening visit. Written informed consent was obtained after thorough explanation of the study to the subjects. Data from a subsample of subjects (14 MtF, 9 FC, and 13 MC) have been published in a previous study (24). The study was approved by the Ethics Committee of the Medical University of Vienna.

### Study Design and Treatment Protocol

The study was designed as a longitudinal monocenter study. Fourteen FtM and 19 MtF transsexuals underwent a baseline scan before start of hormone treatment (PET 1). Scans of 11 FC and 24 MC served as control measurements, while 8 of the 24 MC were measured twice to determine the test-retest reliability of [^11^C]DASB (time interval: 10.13 ± 9.66 days). Baseline scans in FtM and those in FC were performed irrespective of their menstrual cycle phase. A subset of transsexuals underwent a second scan (PET 2; 9 FtM, 16 MtF) 4 weeks after treatment start, and a third scan (PET 3; 11 FtM, 13 MtF) 4 months after treatment start. Hormone treatment followed protocols routinely implemented at the Department of Obstetrics and Gynecology, Unit for Gender Identity Disorder, at the Medical University of Vienna. FtM received 1000 mg testosterone undecanoate every 12 weeks (Nebido 250 mg/mL, 4 mL vial, intramuscular; Bayer, Vienna, Austria). Two cases further received 10 mg to 15 mg lynestrenol (Orgametril 5 mg, oral; Organon, Oss, The Netherlands) daily. MtF received either daily 50 mg cyproterone acetate (Androcur 50 mg tablet, oral; Bayer; 14 subjects) or triptorelin acetate 4.12 mg/month (Decapeptyl 172 mg powder for suspension for injection subcutaneous or intramuscular; Ferring Arzneimittel, Vienna, Austria; 4 subjects). Additionally, MtF over 40 years of age received daily doses of 100 μg estradiol (transdermal therapeutic system applied twice a week; Estradot, Novartis, Vienna, Austria/Estramon, Hexal, Vienna, Austria; 3 subjects), while those less than 40 years of age received 4 mg/day estradiol hemihydrate (Estrofem 2 mg, oral; Novo Nordisk, Vienna, Austria; 9 subjects). Alternatively, six subjects received estradiol hemihydrate .75 mg/day to 1.5 mg/day (Estro-Gel .75 mg/1.25 g/day, transdermal; Meda, Vienna, Austria). Because of extensive hair loss, six MtF further received 2.5 mg/day of the 5-alpha reductase inhibitor finasteride (5 mg, oral; Ratiopharm, Vienna, Austria). One MtF terminated the study prematurely, moved out of town after the baseline PET, and received no medication at our hospital.

### Serum Sampling

Blood samples were collected before PET scanning for transsexuals at each visit. The analysis of plasma levels of estradiol, testosterone, and progesterone was done by the Department of Laboratory Medicine, Medical University of Vienna, Austria (http://www.kimcl.at).

### Positron Emission Tomography

All PET scans were performed in a GE Advance full-ring scanner (General Electric Medical Systems, Milwaukee, Wisconsin) in three-dimensional mode at the Department of Biomedical Imaging and Image-Guided Therapy, Division of Nuclear Medicine, Medical University of Vienna. A 5-minute transmission scan was done using retractable ^68^Ge rod sources for tissue attenuation correction (24, 37). Data acquisition started simultaneously with a bolus injection of [^11^C]DASB measuring brain radioactivity in a series of 50 consecutive time frames. Mean injected dose and specific activity were not significantly different between groups at any time point (*t* test, *p* > .05; Table 1). Total acquisition time was 90 minutes. Collected data were reconstructed in volumes consisting of 35 transaxial sections (128 × 128 matrix) using a fourier rebinning iterative filtered back-projection algorithm with a spatial resolution of 4.36 mm full-width at half maximum 1 cm next to the center of the field of view; for radiotracer preparation and radiochemical variables, see Lanzenberger *et al*. (38) and Haeusler *et al*. (39).

**Table 1:** Plasma Hormone Levels Before, After 4 Weeks, and After 4 Months of Testosterone Treatment in FtM Transsexuals and Antiandrogen and Estrogen Treatment in MtF Transsexuals

	FtM			MtF		
	PET 1	PET 2	PET 3	PET 1	PET 2	PET 3
*n*	14	9	11	19	16	13
T ng/mL	.65 ± .86	3.76 ± 2.06^a^	6.37 ± 2.84^a^	4.38 ± 1.47	.53 ± 1.28^a^	.58 ± 1.37^a^
E_2_ pg/mL	74.00 ± 46.80	83.89 ± 83.0	60.73 ± 25.55	26.68 ± 12.56	82.94 ± 46.07^a^	89.00 ± 62.35^a^
P ng/mL	3.44 ± 5.00	2.83 ± 3.90	.79 ± .38	.61 ± .22	.46 ± .20	.43 ± .16^a^
ID MBq/kg	306.76 ± 45.98	322.15 ± 53.83	333.31 ± 58.82	346.48 ± 66.75	351.43 ± 49.77	345.46 ± 57.61
SA GBq/µmol	13.90 ± 13.33	18.22 ± 13.77	19.83 ± 18.50	25.00 ± 19.97	19.53 ± 17.07	16.84 ± 30.84

Values represent means ± SD. Radiochemical variables injected dose and specific activity were not significantly different between groups at any time point, assessed using *t* tests.

E_2_, estradiol; FtM, female-to-male; ID, injected dose; MtF, male-to-female; P, progesterone; PET, positron emission tomography; SA, specific activity; T, testosterone.

*a* Indicates significant difference from PET 1, *p* < .05 corrected.

### Quantitative Analysis and Regions of Interest

Following between-frame motion correction, individual summed PET images were spatially normalized to a PET template in stereotactic Montreal Neurological Institute space using SPM8 (Wellcome Trust Centre for Neuroimaging, London, United Kingdom; http://www.fil.ion.ucl.ac.uk/spm/). The SERT nondisplaceable binding potential (BP_ND_) (40) was quantified using the multilinear reference tissue model (41). Cerebellar gray matter (excluding vermis and venous sinus) was used as reference region, as postmortem and in vivo SERT quantification identified the cerebellar gray matter as optimal reference region for [^11^C]DASB (42, 43). All modeling calculations were performed using PMOD image analysis software, version 3.3 (PMOD Technologies Ltd, Zurich, Switzerland; www.pmod.com). SERT BP_ND_ was computed in a region of interest (ROI) based approach. Twelve ROIs were selected including the insula; anterior, middle, and posterior cingulate cortex; hippocampus; amygdala; hypothalamus; caudate; putamen; thalamus; and dorsal and median raphe nucleus. ROIs were based on the automated anatomical labeling brain atlas (44), except for dorsal and median raphe nuclei, which were defined manually according to Kranz *et al*. (45). ROIs were selected based on their moderate to high concentration of SERT BP_ND_ (46, 47), the overlap between sex steroid receptor distribution and adequate SERT BP_ND_ (48, 49), and because of gender-specific SERT BP_ND_ abnormalities in mood disorders (13, 15).

### Statistics

Treatment-induced hormone level changes were assessed using linear mixed models analysis with group (FtM, MtF) and time (PET 1–3) as fixed factors and subjects as the random factor, followed by separate models for each group and post hoc pairwise comparisons, corrected for multiple comparisons using the Bonferroni procedure. Similarly, changes in regional SERT BP_ND_ over time were assessed using linear mixed models with group as the between-subjects factor, time and ROI as repeated factors adjusted for baseline values, and subjects as the random factor. Associations between treatment-induced hormonal changes and changes in regional SERT BP_ND_ were calculated using Pearson product-moment correlations. This was followed by exploratory partial correlations to correct for initial hormone level (e.g., testosterone) and for changes of other hormones (e.g., estradiol or progesterone). The significance level was set at 5% in all analyses. SPSS version 19.0 for Windows (SPSS Inc., Chicago, Illinois; www.spss.com) was used for statistical analysis.

## Results

### Hormones

Estradiol, testosterone, and progesterone plasma levels at each time point and plasma level changes between time points were normally distributed for each group (assessed using Kolmogorov-Smirnov test and visual inspection). As expected, androgen treatment in FtM and antiandrogen and estrogen treatment in MtF had profound effects on hormone plasma levels, revealed by a significant interaction group × time (testosterone: *F*_2,38.5_ = 67.74, *p* < .001; estradiol: *F*_2,53.6_ = 5.92, *p* = .005; progesterone: *F*_2,27.2_ = 4.41, *p* = .022). Androgen treatment in FtM led to significant changes in testosterone (*F*_2,14.1_ = 27.94, *p* < .001) with post hoc pairwise comparisons revealing increases from PET 1 to PET 2 (*p* = .005), PET 1 to PET 3 (*p* < .001), and a trend for PET 2 to PET 3 (*p* = .086). Conversely, antiandrogen and estrogen treatment in MtF led to significant decreases of testosterone (*F*_2,26_ = 42.21, *p* < .001), when comparing PET 1 to PET 2 (*p* < .001) and PET 1 to PET 3 (*p* < .001) but not when comparing PET 2 to PET 3 (*p* > .1). Estradiol plasma levels did not change over the course of treatment in FtM (*F*_2,12.81_ = .64, *p* = .541), but they profoundly changed in MtF (*F*_2,17.89_ = 16.90, *p* < .001), showing significant increases from PET 1 to PET 2 (*p* = .001) and PET 1 to PET 3 (*p* = .011) but not from PET 2 to PET 3 (*p* > .1, all *p* corrected). Progesterone plasma levels showed a trend toward decreased values over time in FtM (*F*_2,10.3_ = 3.16, *p* = .085), whereas reductions were significant in MtF (*F*_2,28.4_ = 3.95, *p* = .031) with post hoc tests revealing a significant decrease from PET 1 to PET 3 (*p* = .034, corrected; Table 1).

### SERT BP_ND_

SERT BP_ND_ at each time point and BP_ND_ changes between time points were normally distributed for each region and group. The comparison between transsexuals and healthy control subjects revealed significantly higher SERT BP_ND_ in MC compared with FtM, whereas no other group comparisons showed significant differences (for further information, see Supplement 1). Testing the effect of treatment in transsexuals revealed a significant main effect of time (*F*_2,872.3_ = 12.14, *p* < .001), group (*F*_1,335.6_ = 4.13, *p* = .043), and ROI (*F*_11,854.4_ = 7.09, *p* < .001) as well as significant interactions for time × goup (*F*_2,872.3_ = 19.13, *p* < .001), time × ROI (*F*_22,858.8_ = 3.23, *p* < .001), and time × group × ROI (*F*_22,858.8_ = 2.03, *p* = .004).

**Figure 1:**
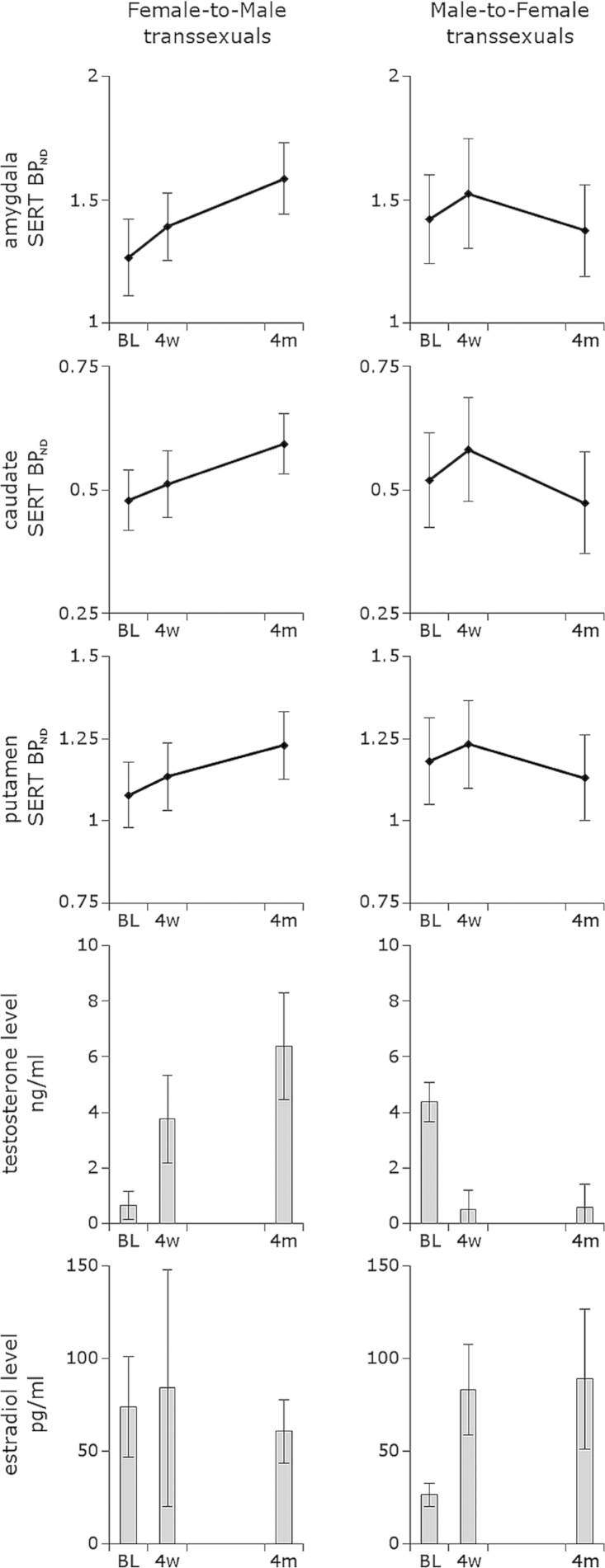
Line chart showing changes in serotonin transporter (SERT) binding potential (BP_ND_) and plasma levels of testosterone and estradiol in female-to-male and male-to-female transsexuals over the course of cross-sex steroid treatment. Depicted are means ± 95% confidence interval at positron emission tomography (PET) 1, i.e., baseline (BL), after 4 weeks (4w) of treatment at PET 2, and after 4 months (4m) of treatment at PET 3.

To interpret the three-way interaction with respect to potential group differences, post hoc two-sample *t* tests for group comparisons were made for each time point and region. This revealed that most regions in MtF had numerically higher BP_ND_ values compared with FtM at all three time points. However, only in the amygdala at baseline, the higher BP_ND_ in MtF was significant at an uncorrected level of *p* = .037. To interpret the three-way interaction with respect to treatment effects, separate models were calculated for each group and region, followed by post hoc pairwise comparisons of the three time points. These analyses revealed a significant increase in SERT BP_ND_ over time in FtM in the amygdala (*p* = .002), caudate (*p* = .002), putamen (*p* = .009), and median raphe nucleus (*p* = .045) and a trend for hippocampus (*p* = .080), thalamus (*p* = .053), and dorsal raphe nucleus (*p* = .053) (Figure 1). However, when correcting for the number of performed tests, only changes in amygdala and caudate remained significant.

In contrast to FtM transsexuals, MtF transsexuals exhibited a decrease of SERT BP_ND_ over the course of 4 months in the insula (*p* = .023) and anterior and mid-cingulate cortex (*p* = .002 and *p* = .015, respectively) and a trend for putamen (*p* = .068), with changes only in anterior cingulate cortex surviving correction for multiple testing (see Table 2 for estimated means ± SE and post hoc pairwise comparisons). Controlling for individualized treatment regimens did not change the main findings (further information can be found in Supplement 1).

**Table 2:** Changes in Serotonin Transporter Binding Potentials over Time in 12 A Priori Regions of Interest in FtM and MtF Transsexuals

	FtM			MtF		
	PET 1	PET 2	PET 3	PET 1	PET 2	PET 3
*n*	14	9	11	19	16	13
INS	.31 ± .04	.33 ± .04	.35 ± .04	.38 ± .03	.41 ± .03	.35 ± .03^c^
ACC	.24 ± .02	.26 ± .02	.25 ± .03	.23 ± .03	.26 ± .03	.20 ± .02^b^^*,*^^c^
MCC	.17 ± .03	.18 ± .03	.17 ± .04	.16 ± .03	.17 ± .03	.12 ± .03^c^
PCC	.10 ± .02	.12 ± .02	.12 ± .02	.07 ± .02	.08 ± .01	.05 ± .01
HIP	.34 ± .04	.38 ± .03	.42 ± .04^a^	.46 ± .03	.48 ± .03	.45 ± .03
AMY	1.27 ± .07	1.39 ± .06	1.58 ± .07^b^^*,*^^c^	1.42 ± .09	1.52 ± .11	1.38 ± .09
HYP	1.91 ± .08	1.94 ± .07	2.18 ± .12^a^	1.93 ± .11	2.04 ± .11	1.87 ± .10
CAUD	.48 ± .03	.51 ± .03	.59 ± .03^b^^*,*^^c^	.52 ± .05	.58 ± .05	.47 ± .05^c^
PUT	1.08 ± .05	1.14 ± .05	1.23 ± .05^a^^*,*^^c^	1.18 ± .06	1.23 ± .06	1.13 ± .06^c^
THAL	1.02 ± .05	1.07 ± .05	1.14 ± .05^a^	1.05 ± .06	1.12 ± .06	1.04 ± .06
DRN	2.63 ± .20	2.55 ± .21	3.33 ± .31^a^^*,*^^c^	3.10 ± .21	3.46 ± .26	3.36 ± .36
MRN	2.13 ± .21	2.51 ± .20^b^	3.09 ± .39^a^	2.48 ± .17	2.90 ± .22	2.64 ± .16

Values are estimations based on the mixed models analyses and represent means ± SE at baseline (PET 1), 4 weeks after (PET 2), and 4 months after (PET 3) start of cross-sex steroid hormone treatment.

ACC, anterior cingulate cortex; AMY, amygdala; CAUD, caudate; DRN, dorsal raphe nucleus; FtM, female-to-male; HIP, hippocampus; HYP, hypothalamus; INS, insular cortex; MCC, middle cingulate cortex; MRN, median raphe nucleus; MtF, male-to-female; PCC, posterior cingulate cortex; PET, positron emission tomography; PUT, putamen; THAL, thalamus.

*a,b* Indicates significant changes from PET 1.

*c,d* Indicates significant changes from PET 2.

*a,c* Post hoc pairwise comparisons are uncorrected.

*b,d* Post hoc pairwise comparisons corrected at *p* < .05.

In the subsample of eight MC, no significant differences were present between the two measurements and test-retest reliability was high (intraclass correlation coefficient > .8; Supplement 1).

### Associations between Hormonal Changes and Changes in SERT BP_ND_

**Figure 2:**
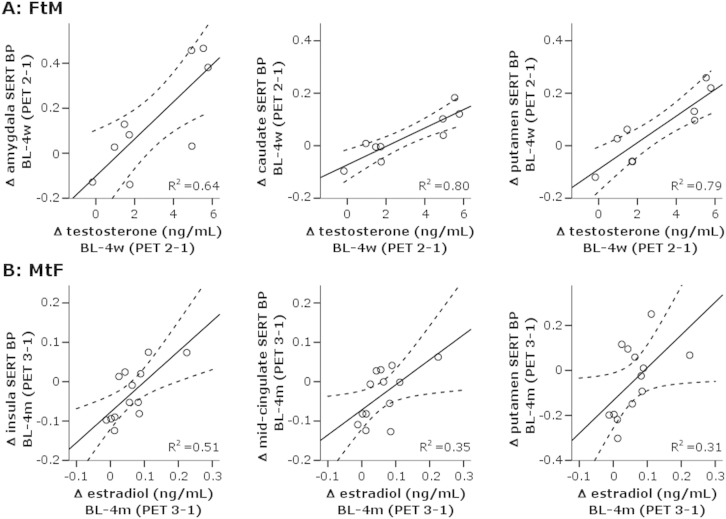
Scatter plots depicting associations between the change in serotonin reuptake transporter (SERT) binding potential (BP) and in plasma steroid hormones for **(A)** female-to-male (FtM) and **(B)** male-to-female (MtF) transsexuals. Scattered lines represent 95% confidence intervals. Positive associations between changes in SERT BP in amygdala, caudate, and putamen and testosterone plasma level increase within the first 4 weeks (4w) of treatment (baseline [BL] − 4w) were found in FtM transsexuals (upper row). Positive associations between changes in SERT BP in insula, mid-cingulate, and putamen and estradiol plasma level increase over the course of 4 months (4m) of treatment (BL − 4m) were found in FtM transsexuals (bottom row). PET, positron emission tomography.

The significant treatment-induced increase in testosterone plasma levels in FtM showed strong positive correlations with changes in regional SERT BP_ND_ after 4 weeks of treatment (PET 1 vs. PET 2; Figure 2A). That is, increases in testosterone plasma levels correlated with increases in SERT BP_ND_ in anterior cingulate cortex (*p* = .027), amygdala (*p* = .010), caudate (*p* = .001), putamen (*p* = .001), and thalamus (*p* = .015) and a trend for median raphe nucleus (*p* = .053). No significant correlations were observed in other regions, indicating a regional effect. Controlling for baseline testosterone levels and changes in estradiol plasma levels in separate partial correlation analyses reduced the *p* value to a nonsignificant trend for the anterior cingulate cortex (*p* = .075 and *p* = .076, respectively) but did not change the significance of results in other regions. Likewise, controlling for changes in progesterone plasma levels had no significant effect. However, correlations between testosterone and SERT BP_ND_ became nonsignificant when correlating hormonal changes with changes in SERT BP_ND_ after 4 months of treatment (PET 1 vs. PET 3).

In contrast to the results in FtM, treatment-induced decreases in testosterone plasma levels and increases in estradiol plasma levels in MtF after 4 weeks of treatment (PET 1 vs. PET 2) were not correlated with changes in SERT BP_ND_ in any of the investigated brain regions. Partial correlation analyses did not change the results. However, correlating significant estradiol changes after 4 months of treatment (PET 1 vs. PET 3) with changes in regional SERT BP_ND_ (PET 1 vs. PET 3) showed positive associations in the insula (*p* = .006), mid-cingulate cortex (*p* = .032), hippocampus (*p* = .048), and putamen (*p* = .046) (Figure 2B). When controlling for progesterone plasma levels, significances increased for insula (*p* = .004) and mid-cingulate (*p* = .026) but decreased to a trend for hippocampus (*p* = .079) and putamen (*p* = .063). Significant correlations were also observed when comparing values after 4 weeks with those after 4 months of treatment (PET 2 vs. PET 3) in insula (*p* = .027) and mid-cingulate cortex (*p* = .020) in regions that also showed a significant decrease in SERT BP_ND_ (Table 2).

Finally, Pearson product-moment correlations revealed no significant associations between depressive symptoms and other clinical characteristics and regional SERT BP_ND_ at any time point (for clinical characteristics, see text and Table S1 in Supplement 1).

## Discussion

In this study, we found that testosterone treatment in FtM transsexuals significantly increased SERT BP_ND_ after 1 month as well as after 4 months of treatment in regions with adequate signal-to-noise ratio, i.e., moderate to high SERT density (Figure 1, Table 2). Early animal studies demonstrated that, in contrast to 5α-dihydrotestosterone, a nonaromatizable androgen, testosterone increased SERT density and SERT messenger RNA in rats (50). While 5α-dihydrotestosterone cannot be converted to estrogen by aromatase, testosterone action is suggested to depend in part on its conversion to estradiol. Furthermore, brain masculinization is believed to depend on organizational effects of estrogen, triggered by aromatized testosterone, to which the initially undifferentiated brain is exposed (51). We therefore suggest that testosterone treatment in FtM affected SERT binding via aromatization to estradiol and activation of estrogen receptors.

Plasma testosterone levels rose progressively over the course of treatment in FtM. Correspondingly, our data show a progressive increase in SERT BP_ND_ from subchronic testosterone administration (4 weeks) to long-term effects (4 months). Furthermore, acute increases in SERT BP_ND_ (after 4 weeks) in anterior cingulate cortex, amygdala, caudate, putamen, and thalamus were found to be positively correlated with changes in plasma testosterone levels. However, no correlations were observed after 4 months of treatment (PET 1 vs. PET 3). If one assumes a causal relationship, this apparent loss of SERT dependency on testosterone levels after 4 months of treatment therefore points to ceiling effects of the influence of testosterone levels.

In contrast to SERT BP_ND_ increases in FtM after testosterone treatment, regional SERT BP_ND_ in MtF remained constant after the first 4 weeks of estrogen and antiandrogen treatment. However, values significantly decreased after 4 months of treatment in anterior and mid-cingulate cortex and insula, whereas hormonal levels significantly changed only within the first 4 weeks after treatment start. Assuming that the effects of testosterone on SERT density depend on its conversion to estradiol, SERT BP_ND_ should remain constant when reducing testosterone levels while increasing those of estradiol, which is in accordance with our data. The significant decrease of regional SERT BP_ND_ after 4 months of treatment, on the other hand, warrants a different explanation that apparently exceeds estradiol effects on SERT BP_ND_. Still, negative associations between estradiol increase and regional SERT BP_ND_ downregulation after 4 months of treatment showed that regional SERT BP_ND_ decreased to a lesser extent when estradiol level increases were larger. In other words, estradiol increases seemed to have a protective effect against SERT BP_ND_ loss.

Our results indicate increases in SERT BP_ND_ when testosterone (and aromatized estradiol) increases. According to a simple mechanistic view on SERT function, elevated SERT availability increases serotonergic uptake, which leads to reduced extracellular serotonin. Furthermore, according to the serotonin hypothesis of depression, serotonin deficiency is considered a hallmark underlying depressive symptoms. Thus, SERT BP_ND_ may be expected to be high in depressed subjects (16, 52, 53), which is, however, in contrast to our meta-analysis showing SERT reductions in several regions (12). Furthermore, studies indicate that testosterone supplementation improves depressive symptoms in hypogonadal men as well as in surgically menopausal women (54, 55), and preliminary data indicate that short-term estradiol treatment may qualify as an effective therapy for perimenopausal major depression (56). Recent animal research indicates that androgen therapy elevates serotonin levels and that this is dependent on aromatase activity (57). Accordingly, exogenous androgen increases aromatization to estradiol, which leads to increases in serotonin synthesis and availability via estrogen receptors (58). Furthermore, according to the use it or lose it hypothesis formulated by Ramamoorthy *et al.* (59), SERT cell surface expression is increased in response to increased synaptic serotonin. Conversely, SERT proteins are downregulated when 5-HT levels are low. Taken together, our data indicate that testosterone treatment in FtM increased serotonin levels, which thereby increased SERT expression. With reference to depression, our interpretations are still very speculative since no associations were found between depressive symptoms and regional SERT BP_ND_. Future research should investigate testosterone-induced changes of serotonergic neurotransmission and the potential benefits of testosterone as add-on therapy to selective serotonin reuptake inhibitor treatment in major depression.

Our study includes limitations that compromise the interpretation of its results. First, our results in transsexual subjects cannot easily be generalized to other human studies. According to Swaab and Garcia-Falgueras (60), transsexuality may derive from a mismatch between sex differentiation of the brain and of the body. Transsexual subjects exhibit features in brain structure and function that reflect their gender identity rather than their genetic sex (24, 61, 62, 63). However, they may also exhibit features that are specific to their condition, which can include the neural underpinnings of well-being, self-esteem, and psychological strain (64, 65, 66). We cannot exclude that these characteristics include alterations within the serotonergic system. However, when comparing transsexuals with their sex-matched control subjects (e.g., FtM with FC and MtF with MC), there was no significant difference in regional SERT BP_ND_. This is in accordance with our previous publication (24), a finding which can be explained by the overlap in study participants between the current and the previous study. On the other hand, in the current study, MC had significantly higher SERT BP_ND_ than FtM in several regions. This is in accordance with Jovanovic *et al.* (19), showing decreased BP_ND_ in women compared with men, but in disagreement with Erritzoe *et al.* (20), showing the opposite pattern in the midbrain.

Second, reductions of testosterone in most MtF are achieved by inhibition of the negative diencephalic pituitary testicular feedback system by the antiandrogen cyproterone acetate. This drug exhibits a close structural relationship with progestogens; therefore, we cannot exclude a more direct effect that goes beyond the modulation of testosterone levels. Indeed, progesterone levels decreased over time in MtF. Still, when correlating changes in testosterone plasma levels with those in SERT BP_ND_, correction for progesterone changes did not influence the results. However, correction affected the relation between estradiol and SERT BP_ND_ changes.

Third, no arterial blood samples were available to quantify SERT BP_ND_ independent of a reference region. However, previous studies investigating SERT occupancy using [^11^C]DASB identified the cerebellar gray matter as optimal reference (42, 52). Even a 50% difference in cerebellar specific binding between subjects would lead to only 3.5% bias for group comparisons in target regions (52).

Finally, since psychiatric disorders other than transsexuality were no exclusion criteria, this could have affected SERT BP_ND_. Indeed, 12 of the 33 transsexuals (4 of 14 FtM and 8 of 19 MtF) were diagnosed with a previous mood or anxiety disorder (i.e., dysthymic disorder, major depressive disorder in full or partial remission, or social phobia) according to the Structural Clinical Interview for DSM-IV Disorders. However, comparing SERT BP_ND_ in subjects with and without a previous diagnosis, groups did not significantly differ.

In conclusion, our data provide evidence that long-term high-dosage cross-sex steroid hormone treatment in transsexual subjects affects SERT binding in a hormone-specific manner. Testosterone treatment increased SERT binding, whereas chronic antiandrogen treatment decreased it. Given the central role of the SERT in the treatment of depression and anxiety disorders, our findings may lead to new treatment modalities and understanding of the mechanism of action of antidepressant treatment properties.
