# Supplementary material for: High-Dose Testosterone Treatment Increases Serotonin Transporter Binding in Transgender People
Source: Biol Psychiatry. 2015 Oct 15;78(8):525–33. doi: 10.1016/j.biopsych.2014.09.010 (PMC4585531; doi:10.1016/j.biopsych.2014.09.010)
Supplement: Supplementary file 1 — Supplementary Material [file mmc1.pdf]

High-Dose Testosterone Treatment Increases Serotonin Transporter Binding in Transgender People

Supplemental Information

Comparison between Transsexuals and Healthy Controls

Comparisons between transsexuals and healthy controls at baseline were done using mixed models analysis with Group (FtM, MtF, MC, FC) as fixed factor, ROI as repeated factor and Subjects as random factor. Linear mixed models analysis revealed a main effect of Group ($F_{3,161.6} = 17.26$, $p < 0.001$) and ROI ($F_{11,103.3} = 290.39$, $p < 0.001$) and a significant interaction between Group and ROI ($F_{33,103.3} = 1.80$, $p = 0.014$). Post-hoc two-sample *t*-tests for each region revealed significantly higher SERT BP_{ND} in MC compared to FtM in the insula ($p = 0.019$), hippocampus ($p = 0.032$), amygdala ($p < 0.001$), caudate ($p = 0.017$), putamen ($p = 0.005$) and dorsal raphe nucleus ($p = 0.009$). In the dorsal raphe nucleus, MC SERT BP_{ND} was also higher compared to MtF ($p = 0.037$). When correcting for multiple comparisons using the Bonferroni procedure, differences remained significant only for the amygdala. No other group comparisons were significant although most regions in MtF had numerically higher BP_{ND} values compared to FtM (see Table 1).

Testing the test-retest reliability in the subsample of 8 MC revealed high repeatability both in cortical (intra-class correlation coefficient [ICC]: insula = 0.84, anterior cingulate cortex = 0.95, middle cingulate cortex = 0.90) and subcortical regions (e.g., caudate = 0.90, putamen = 0.81, thalamus = 0.84). The ICC reflects the ratio of the between subjects variance to total variance, i.e., the stability of the measurement between time point 1 and time point 2 and was calculated from the results of a two-way ANOVA

model with subjects treated as random effects and sessions treated as fixed effects. A paired *t*-test for each ROI in these subjects revealed no significant difference between the two measurements. Interestingly though, no significant differences were observed when comparing the significant SERT BP_{ND} changes in FtM to the non-significant changes in MC. However, comparing the changes in MC with those in MtF revealed a significant difference in the insula (p = 0.02), anterior (p = 0.02), mid- (p = 0.02) and posterior cingulate (p = 0.003, uncorrected).

Testing Potential Confounders of Treatment Effects

Excluding the two FtM subjects receiving lynestrenol did not change the main findings but slightly increased *p* values for amygdala and median raphe nucleus and reduced *p* values, i.e., increased significances in hypothalamus, caudate, putamen and thalamus.

Including the type of anti-androgen treatment (cyproterone acetate vs. triptorelin acetate) in MtF as factor did not change the significance of main results. In no ROI was there a significant interaction between drug type and time point. Similarly, including finasteride intake as factor slightly increased *p* values for insula, anterior and mid-cingulate but decreased *p* values for caudate. In no ROI was there a significant interaction between drug type and time point.

When investigating a potential effect of an additional DSM-diagnosis on baseline SERT BP_{ND} in transsexuals, neither the main effect nor the interaction with group was revealed to be significant.

Clinical Characteristics

Transsexuals completed the 21-item Hamilton Depression Rating Scale (HAM-D), the Hamilton Anxiety Rating Scale (HAM-A), a German inventory for the assessment of factors of aggression (FAF, Fragebogen zur Erfassung von Aggressivitätsfaktoren) (1) and the World Health Organization Quality of Life instrument WHOQOL-BREF (2) at each scanning day.

Scores for each group and at each time point were normally distributed. For HAM-D and FAF, mixed models analysis revealed no significant main effect of Group or Time and no interaction effect ($p > 0.1$). The subscale autoaggression of FAF, however, was lower in FtM than MtF, as revealed by a significant main effect of Group ($F_{1,69.1} = 5.82$, $p = 0.018$). Furthermore, there was a significant change in HAM-A scores over time as indicated by a significant main effect of Time ($F_{2,54.3} = 9.31$, $p < 0.001$), with post-hoc *t*-tests revealing reductions from one month to four months after treatment start (PET 2 to PET 3) in MtF ($p = 0.011$, corrected) and a trend in FtM ($p = 0.058$, corrected). Finally, for the quality of life social relationships domain, there was a main effect of Group ($F_{1,57.3} = 6.02$, $p = 0.017$) showing higher values in FtM compared to MtF (see Table S1). However, in all other domains and in the global score of the WHOQOL-BREF there was no significant difference. Finally, Pearson product-moment correlations revealed no significant associations between clinical characteristics and regional SERT BP_{ND} at each time point.

Table S1. Clinical variables are given in means and SD per measurement time and group. Clinical variables included the Hamilton Depression Rating Scale (HAM-D), the Hamilton Anxiety Rating Scale (HAM-A), the Factors of Aggression inventory (FAF) and the WHO Quality of Life Questionnaire - Short Version (WHOQOL-BREF).

	**FtM**			**MtF**		
	**PET 1**	**PET 2**	**PET 3**	**PET 1**	**PET 2**	**PET 3**
n	14	9	11	19	16	13
HAM-D	2.0±3.6	1.6±2.2	1.4±2.3	1.8±1.9	3.3±3.5	1.1±1.8
HAM-A	1.6±2.7	2.8±3.9	0.9±0.9	1.5±1.9	3.4±4.2	1.1±1.5
FAF#	8.1±8.1	6.9±8.6	7.8±9.4	6.9±5.4	6.1±5.5	6.1±4.7
FAF autoaggression	2.7±2.9	2.1±2.7	2.7±3.4	4.1±3.1	4.3±2.8	3.9±2.4
WHOQOL-BREF						
Physical Domain	84.7±15.1	82.5±13.1	85.7±11.0	79.7±16.1	75.7±16.3	79.7±13.5
Psychol. Domain	62.8±19.4	75.0±13.3	70.4±13.4	69.5±16.8	68.2±14.3	74.0±14.8
Social Relations D.	73.8±16.3	79.6±16.0	79.6±16.7	62.7±20.3	65.6±20.9	69.9±26.7
Environmental D.	77.9±14.9	78.7±8.6	81.6±9.3	76.8±15.5	75.4±14.6	76.7±17.1
Global	78.6±13.4	78.4±14.9	80.6±15.5	73.7±15.5	75.0±20.1	73.1±17.6

#sum score of the sub-scales spontaneous aggression, reactive aggression and impulsiveness. None of these sub-scales alone showed significant differences.
D., domain; FtM, female-to-male; MtF, male-to-female; PET, positron emission tomography.

Supplemental References

1. Hampel R, Selg H (1975): *FAF-Fragebogen zur Erfassung von Aggressivitätsfaktoren, Handanweisung*. Gottingen: Hogrefe Verlag für Psychologie.

2. The_WHOQOL_Group (1998): Development of the World Health Organisation. WHOQOL-BREF quality of life assessment. *Psychol Med*. 28:551-558.
